# Supplementary material for: Functional Architecture of the Human Hypothalamus: Cortical Coupling and Subregional Organization Using 7-Tesla fMRI
Source: ArXiv. 2025 Jun 6:arXiv:2506.06191v1. Preprint. [Version 1] (PMC12156108)
Supplement: Supplementary file 1 [file NIHPP2506.06191v1-supplement-1.pdf]

Supplementary Figure

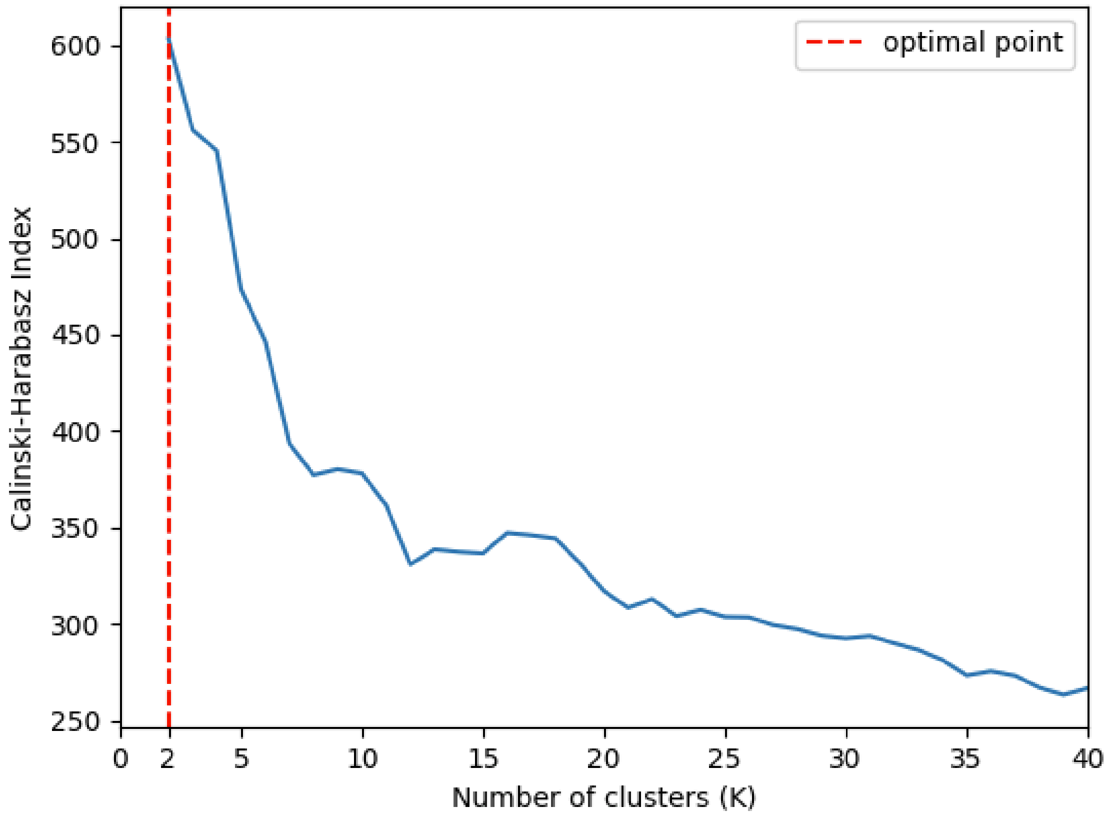

Supplementary Figure 1. Elbow plot of optimal number of clusters (Cortical Networks). The plot shows the Calinski-Harabasz index score on the Y-axis as the number of clusters (Cortical Networks) increases on the X-axis. Higher index scores reflect better clustering, as defined by greater between cluster variances vs. within cluster variance. The optimal number of clusters identified by the Kneedle algorithm was two, indicated by the red dashed line.
